# Supplementary material for: Urinary 15-F2t-Isoprostane Concentrations in Dogs with Liver Disease
Source: Vet Sci. 2023 Jan 21;10(2):82. doi: 10.3390/vetsci10020082 (PMC9958836; doi:10.3390/vetsci10020082)
Supplement: Supplementary file 1 [file vetsci-10-00082-s001.zip › Figure S3.pdf]

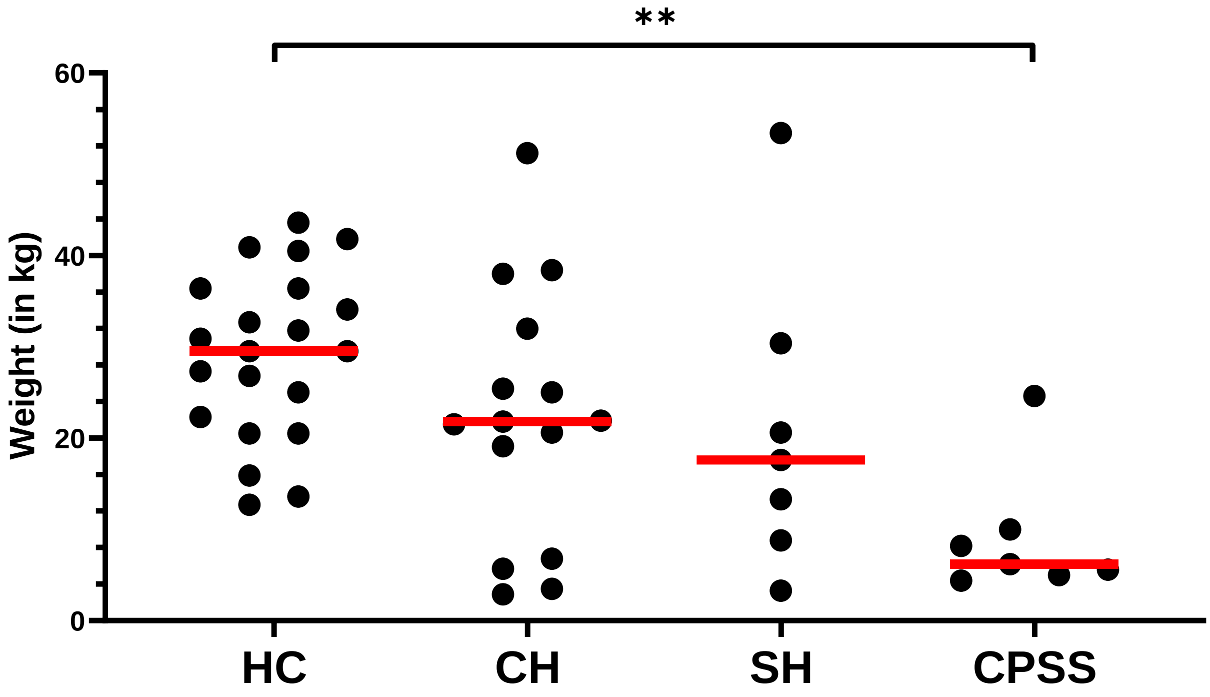

Supplemental Figure S3. Weight of study dogs across groups. Median weight of each group indicated by red line. Asterisks (**) represent a significant difference at $P < 0.01$. Abbreviations: HC, healthy controls; CH, chronic hepatitis; SH, steroid hepatopathy; CPSS, congenital portosystemic shunt.
