# Supplementary material for: Urinary 15-F2t-Isoprostane Concentrations in Dogs with Liver Disease
Source: Vet Sci. 2023 Jan 21;10(2):82. doi: 10.3390/vetsci10020082 (PMC9958836; doi:10.3390/vetsci10020082)
Supplement: Supplementary file 1 [file vetsci-10-00082-s001.zip › Figure S4.pdf]

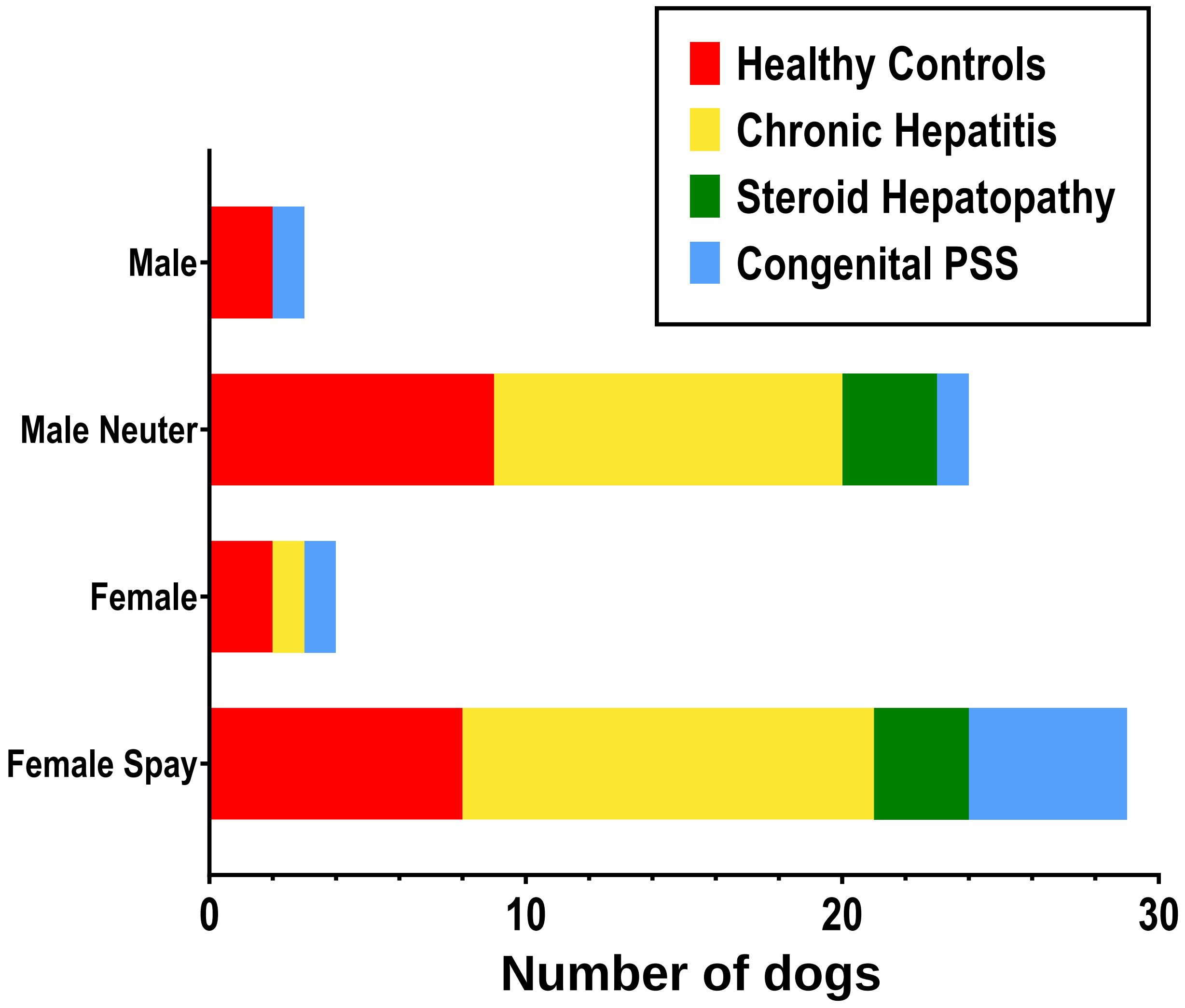

Supplemental Figure S4. Representation of the number of study dogs of each sex and their distribution by cohort. No significant difference was observed among groups ($P = 0.487$).
