# Supplementary material for: Urinary 15-F2t-Isoprostane Concentrations in Dogs with Liver Disease
Source: Vet Sci. 2023 Jan 21;10(2):82. doi: 10.3390/vetsci10020082 (PMC9958836; doi:10.3390/vetsci10020082)
Supplement: Supplementary file 1 [file vetsci-10-00082-s001.zip › Figure S5.pdf]

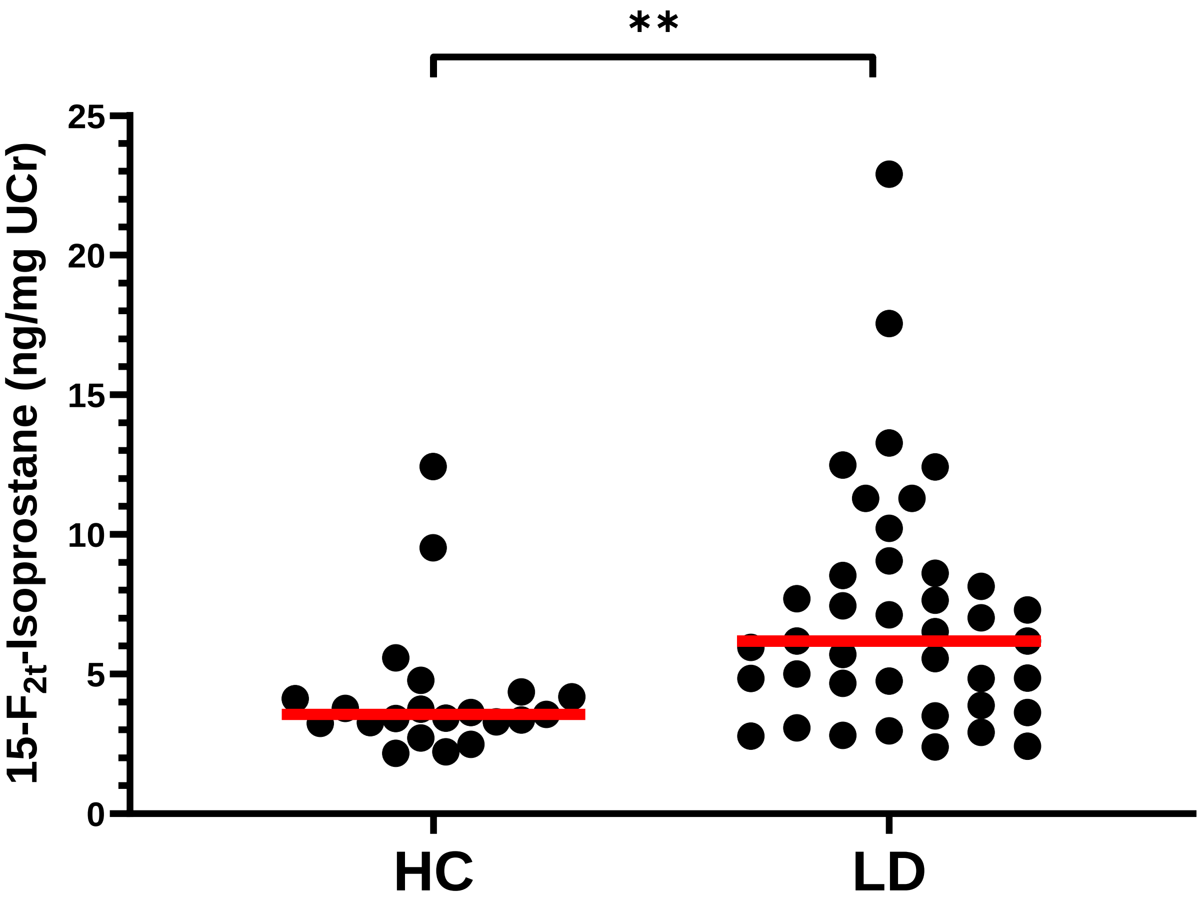

Supplemental Figure S5. Concentrations of 15-F_{2t}-isoprostane (ng/mg urinary creatinine) in the urine of healthy control (HC) dogs and single, combined group of dogs with liver disease (LD). Median of each group is indicated by red line. Asterisks (**) represent a significant difference at $P < 0.01$.
