# Supplementary material for: Urinary 15-F2t-Isoprostane Concentrations in Dogs with Liver Disease
Source: Vet Sci. 2023 Jan 21;10(2):82. doi: 10.3390/vetsci10020082 (PMC9958836; doi:10.3390/vetsci10020082)
Supplement: Supplementary file 1 [file vetsci-10-00082-s001.zip › Figure S6.pdf]

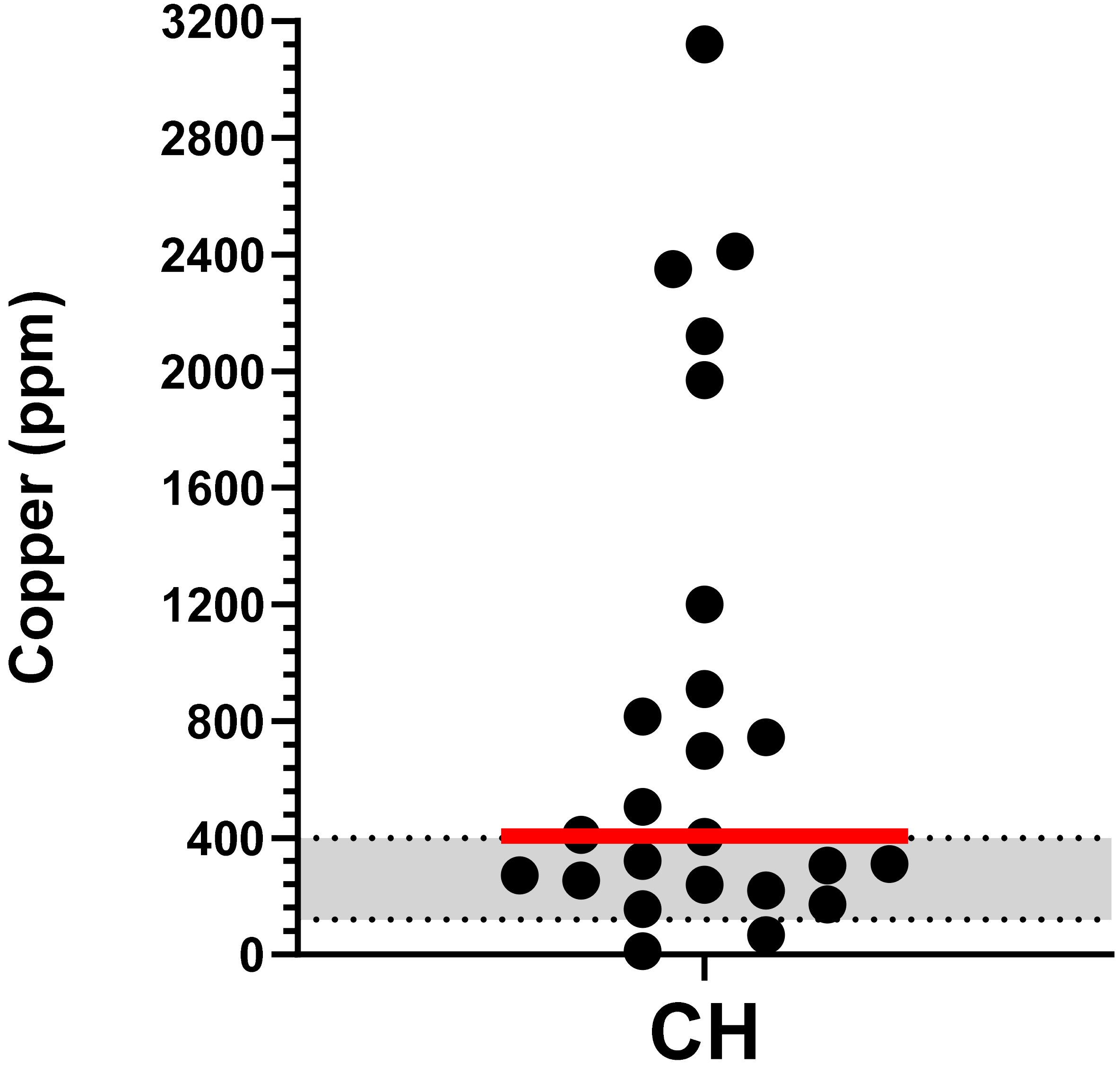

Supplemental Figure S6. Tissue copper quantification from liver biopsies taken from dogs with chronic hepatitis (CH). Median copper is indicated by red line. Gray area represents normal tissue copper concentration range (120 – 400 ppm).
