# Supplementary material for: Urinary 15-F2t-Isoprostane Concentrations in Dogs with Liver Disease
Source: Vet Sci. 2023 Jan 21;10(2):82. doi: 10.3390/vetsci10020082 (PMC9958836; doi:10.3390/vetsci10020082)
Supplement: Supplementary file 1 [file vetsci-10-00082-s001.zip › File S1.pdf]

File S1 -- Urinary 15-F2t-isoprostane concentration (ng/mg urinary creatinine)

as measured by liquid chromatography / negative ion chemical ionization mass spectroscopy at the Eicosanoid Core Laboratory at Vanderbilt University (Nashville, TN)

**Healthy Controls (HC)**	**Chronic Hepatitis (CH)**	**Steroid Hepatopathy (SH)**	**Congenital Portosystemic Shunt (CPSS)**
9.52	6.19	7.70	22.90
12.43	7.45	5.96	17.55
2.72	8.53	8.61	13.28
2.22	2.81	3.88	2.92
3.77	4.86	3.07	12.42
3.29	10.22	4.84	11.29
4.78	6.52	2.39	12.49
3.41	8.15		7.30
3.42	5.55		
3.24	3.51		
4.19	9.06		
3.74	4.75		
5.58	6.18		
2.17	2.78		
4.36	7.02		
3.26	4.67		
3.56	7.65		
2.48	2.97		
4.12	11.29		
3.63	2.42		
3.36	5.00		
	4.85		
	7.13		
	3.62		
	5.71		
