# Supplementary material for: Urinary 15-F2t-Isoprostane Concentrations in Dogs with Liver Disease
Source: Vet Sci. 2023 Jan 21;10(2):82. doi: 10.3390/vetsci10020082 (PMC9958836; doi:10.3390/vetsci10020082)
Supplement: Supplementary file 1 [file vetsci-10-00082-s001.zip › File S2.pdf]

File S2 -- Sample preparation and acquisition parameters for LC-MS-based analysis of urinary 15-F_{2t}-isoprostane concentrations

Analyte	Internal Std	Q1	Q3	Cone	CE	Dwell time (s)	Recovery	%CV
2,3-dinor-15-F2t-IsoP	15-F2t-IsoP-d4	325.1	237.1	40	24	0.022	97%	18.1
2,3-dinor-5,6-dihydro-15-F2t-IsoP	15-F2t-IsoP-d4	327.1	283.1	40	24	0.022	97%	5.65
PGF2a and 15-series-F2-IsoPs	15-F2t-IsoP-d4	353.1	193.1	40	24	0.022	96%	11.8
5-series-IsoPs	15-F2t-IsoP-d4	353.102	115.1	40	24	0.022	95%	13.5
15-F2t-IsoP-d4		357.1	197	40	24	0.022		

Sample Preparation

A stock solution of the isotopically-labeled internal standard ([$^2H_4$]-15-F_{2t}-IsoP, Item No. 316350, Cayman Chemical, Ann Arbor, MI USA) was prepared in ethanol. The concentration of the stock solution was determined by comparison with the 15-F_{2t}-IsoP MaxSpec© standard (Item No. 25903, Cayman Chemical, Ann Arbor, MI USA).

Urine was thawed on ice. A 0.200mL aliquot was diluted with 0.400mL of a solution of 0.1% formic acid/methanol (95/5, v/v) and 0.040mL 0.1N HCl. The solution was vortexed to mix and to [$^2H_4$]-15-F_{2t}-IsoP (1.8ng) was added to each sample. The samples were again mixed and the pH of each was adjusted to pH3 with 0.1N HCl, if required.

The samples were purified by extraction on a Waters HLB 96-well micro-elution plate (Waters Corporation, Milford, MA USA). Sample wells were first washed with methanol (0.200mL x 2) followed by 25% methanol in water (0.200mL x 2). The sample was then loaded on the matrix and washed with 0.400mL of a solution of 0.1% formic acid/methanol (95/5, v/v). The wells were then washed with 0.200mL hexanes. Isoprostanes and metabolites were eluted from the plate with 0.030mL 2-propanol/acetonitrile (50/50, v/v) into a 96-well collection plate containing 0.030mL water in each well.

Samples were analyzed on a Waters Xevo TQ-XS triple quadrupole mass spectrometer connected to a Waters Acquity I-Class UPLC (Waters Corp., Milford, MA USA). Separation of analytes was obtained using a Waters BEH C18 UPLC column (1.0 x 100mm, 1.8μm) with mobile phase A being 0.01% formic acid in water and mobile phase B acetonitrile. Isoprostanes and metabolites were separated using a gradient elution beginning with 20% B going to 95% B at a flow rate of 0.100mL/min (see Gradient Table). The mass spectrometer was operated using multiple reaction monitoring (MRM) in the negative ion mode with argon as the collision gas. Analyte quantification was carried out based on the ratio of the analyte to internal standard peak height. Instrument control and data acquisition utilized MassLynx V4.2; integration and quantitation used TargetLynx 4.2.

The limit of detection is 0.050 ng/mL.

Mass Spectrometer Settings:

Waters Xevo TQ-XS Triple Quadrupole MS

**Operation Mode**	ESI negative
**Capillary**	1 kV
**Desolvation Temp**	500 V
**Desolvation Gas**	1000 L/hr
**Cone Gas**	150 L/hr
**Nebuliser**	7 bar

UPLC Conditions:

Waters Acquity I-Class UPLC

Column: Waters Acquity BEH C18, 1.0 x 100mm, 1.8mm

**Column Temp**	35°C
**Sample Temp**	8°C
**Inj Mode**	Partial Loop
**Inj Loop**	10mL
**Inj Vol**	5mL

Gradient Table

Time (min)	Flow Rate (mL/min)	%A	%B	Curve
Initial	0.100	80	20	Initial
0.10	0.100	80	20	6
5.00	0.100	60	40	6
6.00	0.100	5	95	6
8.50	0.100	5	95	6
8.60	0.100	70	30	6
11.00	0.100	70	30	6
