# Supplementary material for: Urinary 15-F2t-Isoprostane Concentrations in Dogs with Liver Disease
Source: Vet Sci. 2023 Jan 21;10(2):82. doi: 10.3390/vetsci10020082 (PMC9958836; doi:10.3390/vetsci10020082)
Supplement: Supplementary file 1 [file vetsci-10-00082-s001.zip › Table S1.pdf]

Supplementary Table S1. Median concentrations of 15-F_{2t}-isoprostane (ng/mg UCr) in the urine of healthy dogs and dogs with liver disease.

Cohort	Median [Range]
HC	3.6 [2.2 – 12.4]
CH	5.7 [2.4 – 11.3]
SH	4.8 [2.4 – 8.6]
CPSS	12.5 [2.9 – 22.9]
LD	6.2 [2.4 – 22.9]

Abbreviations: HC, healthy controls; CH, chronic hepatitis; SH, steroid hepatopathy; CPSS, congenital portosystemic shunt; LD, combined group of dogs with liver disease (CH + SH + CPSS); UCr, urinary creatinine.
