# Supplementary material for: Urinary 15-F2t-Isoprostane Concentrations in Dogs with Liver Disease
Source: Vet Sci. 2023 Jan 21;10(2):82. doi: 10.3390/vetsci10020082 (PMC9958836; doi:10.3390/vetsci10020082)
Supplement: Supplementary file 1 [file vetsci-10-00082-s001.zip › Table S2.pdf]

Supplementary Table S2. *P*-values of all comparisons of urinary 15-F_{2t}-isoprostane concentrations between groups.

**Single comparison**	***P*-value**
HC vs. LD	.0010
**Dunn's multiple comparisons**	**Adjusted *P*-value**
HC vs. CH	.0821
HC vs. SH	> .9999
HC vs. CPSS	.0004
CH vs. SH	> .9999
CH vs. CPSS	.1414
SH vs. CPSS	.1320

Abbreviations: HC, healthy controls; CH, chronic hepatitis; SH, steroid hepatopathy; CPSS, congenital portosystemic shunt; LD, combined group of dogs with liver disease (CH + SH + CPSS).
